# Supplementary material for: Chromothripsis during telomere crisis is independent of NHEJ, and consistent with a replicative origin
Source: Genome Res. 2019 May;29(5):737–49. doi: 10.1101/gr.240705.118 (PMC6499312; doi:10.1101/gr.240705.118)
Supplement: Supplemental Material [file supp_gr.240705.118_Supplemental_file_1.zip › contigs/annotated_contigs/DB116/contig.2.DB116_length_621_mean_cov_7.48792270531.docx]

**DB116_length_621_mean_cov_7.48792270531**

```
AAAAAATGAAAAAAGTGTTTTCTCTAAGGAAATCCATTTTACAAGTGAAAATTTCTTTGTCCAAGTTCTCTCTATCAGGAGTAATTTAA
 >chr5:123511122-123511306 + E=5e-99 p=0e+00
TTTTCTTCATAATTTAGGCAGAAATAAGTAAGTTTTTCTTGGATTCAAACATCAGCAAGCAATACAAAGACATTATTAGTATGTTTTGT

AATCTT|ATTTTATTATTATTTTAATATAATATTA|TTATTTTAATATAATAATATGTGTTCATTTAAATAAGACCAAATTTCTTCAGG
                                   >chr5:123512980-123513389 + E=2e-234
ATCCTTCAGGAAAGAATAAAGTAAGTATGTCTCCTCTTTGAATATTCCCAAATTATTAATAATAAATTATTTCCTGATGCCAGTTTTAG

TCTTTAGATTCTAAGATTTTACATTAGATGAAATATAAATACTAATTGGAATATTATTGATCTTTTTCAACTTCCTCCTCATAGTAGCT

TCAATAAGCATATTATTATACTAGATAACTTGAAAAAGCATCTCATTTTCTTTGACATTTTTTCTGTTACTTATATCTAGATCACAAAA

TCACACAAGTCTTGGCAAAGAAAGTTTTGAAACTTTGTGGAAGTTTTTGGCATTATCTATGTCATACTTGAAAATCATTTGTTTTAGTC
```
